# Supplementary material for: A functional comparison of two transplantable syngeneic mouse models of melanoma: B16F0 and YUMM1.7
Source: Biol Open. 2025 Sep 11;14(9):bio062175. doi: 10.1242/bio.062175 (PMC12452060; doi:10.1242/bio.062175)
Supplement: Supplementary information [file biolopen-14-062175-s1.pdf]

Table S1. List of fluorescently-conjugated antibodies using to quantify cell subsets by flow cytometry.

Marker	Clone	Fluorophore	Dilution	Manufacturer
LIVE/DEAD Fix	--	Violet/Pacific Blue	--	Invitrogen
CD45	30-F11	BB515	1:80	BD Biosciences #564590
CD3e	500A2	Alexa Fluor 700	1:50	BioLegend #152316
CD4	GK1.5	APC-Cy7	1:80	BD Biosciences #552051
CD8a	REA601	APC	1:50	Miltenyi 130-109-248
CD161 (NK-1.1)	PK136	APC-Cy7	1:20	BioLegend #108723
CD45R/B220	RA3-6B2	APC	1:80	BioLegend #103212
CD49b	DX5	PerCP/Cy5.5	1:80	Biolegend #108915
CD11b	M1/70	PerCP/Cy5.5	1:80	eBioscience #45-0112-80
CD11c	N418	PE	1:40	eBioscience #12-0114-81
F4/80	BM8	APC-Cy7	1:20	BioLegend #123117
Ly-6G/Ly-6C (Gr-1)	RB6-8C5	APC	1:80	BioLegend #108412
CD279 (PD-1)	REA802	PE	1:20	BioLegend #135205
I-A/I-E (MHC-II)	M5/114.15.2	Alexa Fluor 700	1:200	BioLegend #107622
